# Sequence Conservation, Radial Distance and Packing Density in Spherical Viral Capsids

**DOI:** 10.1371/journal.pone.0132234

**Published:** 2015-07-01

**Authors:** Chih-Min Chang, Yu-Wen Huang, Chi-Wen Lee, Tsun-Tsao Huang, Chung-Shiuan Shih, Jenn-Kang Hwang

**Affiliations:** 1 Institute of Bioinformatics and Systems Biology, National Chiao Tung University, HsinChu 30050, Taiwan, R.O.C; 2 Department of Bioinformatics and Medical Engineering, Asia University, Taichung City 41354, Taiwan, R.O.C; Centro Nacional de Biotecnologia (CNB-CSIC), SPAIN

## Abstract

The conservation level of a residue is a useful measure about the importance of that residue in protein structure and function. Much information about sequence conservation comes from aligning homologous sequences. Profiles showing the variation of the conservation level along the sequence are usually interpreted in evolutionary terms and dictated by site similarities of a proper set of homologous sequences. Here, we report that, of the viral icosahedral capsids, the sequence conservation profile can be determined by variations in the distances between residues and the centroid of the capsid – with a direct inverse proportionality between the conservation level and the centroid distance – as well as by the spatial variations in local packing density. Examining both the centroid and the packing density models against a dataset of 51 crystal structures of nonhomologous icosahedral capsids, we found that many global patterns and minor features derived from the viral structures are consistent with those present in the sequence conservation profiles. The quantitative link between the level of conservation and structural features like centroid-distance or packing density allows us to look at residue conservation from a structural viewpoint as well as from an evolutionary viewpoint.

## Introduction

Structural biologists have long used the so-called *radius-colored surface model* in 3D graphics to render the structures of icosahedral capsids of viruses[1–10]. In this model, the residues on the virus surface are color-coded radially from the centroid of the capsid. In this way, interesting surface patterns emerge on capsids. These surface patterns are often indicative of biological functionality, such as the surface pores associated with the export/import of genetic material or molecular components [2,5–7] or surface clefts involved in specific binding. This model can even be applied to low-resolution surface models derived from cryo-EM maps[3,4] to revealing otherwise indiscernible surface patterns. But, the radial-colored surface model is often considered merely as an image enhancement tool without any biological bases.

We examine a set of nonhomologous crystal structures of 51 icosahedral capsids, computing the centroid distances of residues and looking for possible biological connections. We find that, for each capsid, the residues with a similar level of conservation often arrange themselves equidistantly from the centroid of the capsid, with the conservation level of residues in direct inverse proportion to their centroid distances. Hence, a straightforward inference from this is that highly conserved residues tend to lie near the centroids of structures. Indeed, a number of studies[11,12] reported that the catalytic residues (hence, highly conserved) of enzymes are usually found near the centroids of structures. However, the centroid model, being an isotropic model (i.e., free of orientation parameters), is considered as a specialized model for highly symmetrical structures like spherical capsids. Hence, we examine another general model developed by Hwang and co-workers[13,14] on the same dataset. In this model, the sequence conservation profile is modeled by the spatial variations in local packing density. We found that both models perform well on the capsid dataset, though, with regards to accuracy, the packing density model is superior to the centroid model.

The quantitative link between the conservation and structural features found here indicates that the level of amino acid conservation may be derived from atomic coordinates. This is not as surprising as first thought. Structural features (like packing density) have long been recognized related to sequence conservation–a low-density site may accommodate a variety of alternative residue types, whereas a high-density site might be allowed only for a few closely similar residue types. But this observation was considered as of a *qualitative* nature[15] until recently[14,16–18]. Considering that a protein sequence evolves millions of years under the constraints imposed by protein structure and function[19]. It is noted that the amino acid types at certain sites are limited by functional constraints–for example, of catalytic residues in the active sites of enzymes, more than 90% are either charged or polar[20,21]. But since protein function requires a properly folded structure, it is the interactions within the protein structure that will determine residue conservation. Therefore, some structural features of folded proteins may quantitatively reflect residue conservation.

## Materials and Methods

### The packing density model

The packing density of a residue in a protein structure is defined as the number of neighboring residues within a certain cutoff radius. That number is referred to as *contact number* of that residue. Each neighboring residue is counted as contributing a unit of contact number without distance-dependence as long as that residue is within the cutoff distance. Conventionally, the packing density uses only Cα atoms. In other word, the residue is reduced to a Cα atom. Here, we will use the packing density model developed by Lin et al.[22]. This model does not have a cutoff radius. Due to this, it has found a number of interesting applications[21,23,24]. The packing density *n*_*i*_ of residue *i* is defined as

$$n_{i}=\sum_{j\neq i}^{N}1/r_{ij}^{2},$$ (1)

where *r*_*ij*_ is the distance between residue *i* and *j*. It takes into account *all* other residues in the protein, with the unit contribution from each of the other residues weighted by the inverse square of the separation between it and the said residue. Following the convention of Lin et al.[22], we will prefer to use the reciprocal of *n*_*i*_, i.e., $w_{i}=n_{i}^{-1}$. *w*_*i*_ is called the *weighted contact number* (WCN). The *normalized* WCN *z*_*w*_ is defined $z_{w}=(w-\bar{w})/\sigma_{w}$, where $\bar{w}$ and *σ*_*w*_ are the mean and the standard deviation of *w*, respectively. The WCN value is between -1 and 1. Note that the WCN is defined reciprocally to the convention contact number. Hence, the value of -1 gives the highest packing density in a protein and the value of 1 gives the lowest packing density. In the text, the normalized WCN will be simply referred to as the WCN, unless otherwise specified. The WCN profile (or the packing density profile) refers to the series, 〈*z*_*w*,1_,*z*_*w*,2_,*z*_*w*,3_,…,*z*_*w*,*N*_〉. For each subunit of the capsid, its WCN profile is computed taking into account the whole structure of the capsid. In other word, the WCN profile of a subunit includes packing contributions from that subunit as well as from other subunits of the capsid.

### The centroid model

The centroid-distance (or c-distance) of a residue is defined as the distance from the Cα atom of the residue to the centroid of the capsid. The centroid is the average position of the Cα atoms. The centroid of the capsid is computed taking into consideration the whole capsid. For convenience, the c-distance of a residue is normalized in such a way that its average is zero and its standard deviation is 1. The normalized c-distance is denoted as *z*_*r*_. In the text, we will refer to the normalized centroid distance simply as the centroid distance or the radial distance, unless otherwise specified. The distribution of *z*_*r*_ along the polypeptide chain is called *the c-distance profile*.

### Sequence conservation profiles

The site-specific substitution rates are computed by Rate4Site[25] implemented in the ConSurf server[26]. Rate4Site models the evolutionary process considering the phylogenetic relationships between homologous sequences, thus avoiding uneven sampling in sequence space; in addition, it takes into account the stochastic nature of the underlying evolution process, using a probability model based on the JTT matrix[27] to compute amino acid substitution probabilities for each branch in the phylogenetic tree. Following the convention of the work by Shih et al.[13], we will refer to the site's substitution rate as its conservation score; hence, the site of a lower conservation score is more conserved than that of a higher conservation score. In addition, we will refer to the conservation scores along the sequence as the sequence conservation (SC) profile of the protein. To reduce noise, the profile is smoothed using a sliding window as described by Shih et al.[13]. The SC profile is normalized such that its average is zero and its standard deviation is 1, as in the case of the WCN profile described before.

### The Dataset

We selected 51 capsids from the VIrus Particle ExploreR database (VIPERdb)[28] with a sequence homology < 30% of sequence identity. Of the set of capsids, 34 are homomers and 17 are hetereomers. The 51 capsids are listed in S1 Table.

## Results

### Structural features and the levels of conservation for capsid

**Fig 1:**
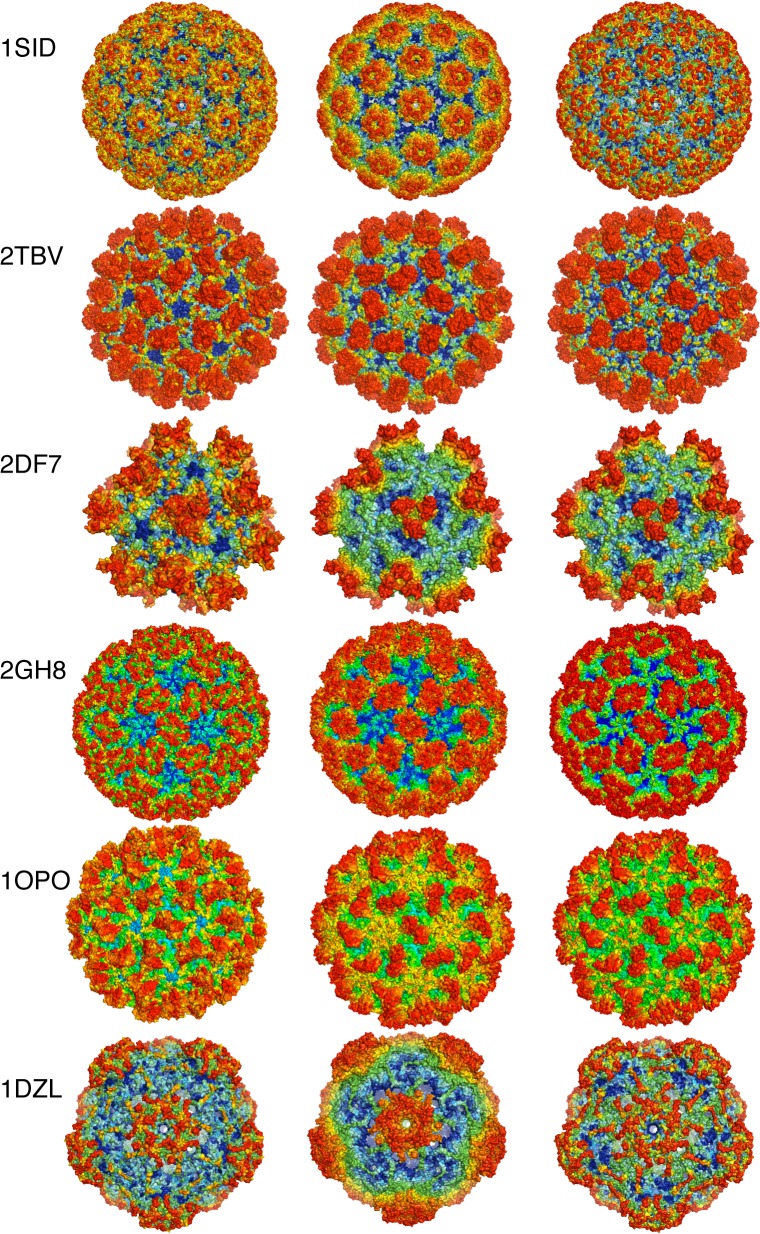
The capsid crystal structures of 6 viruses. Each structure is represent by three surface representations: the conservation-colored structure is shown on the left, the c-distance-colored structure in the middle, and the WCN-colored structure on the right. All structures follow a continuous rainbow color scheme ranging from red to blue, with the red designating the residue that is the farthest from the centroid, the least packed or the least conserved, the blue designating the residue that is closest to the centroid, or the most packed, or the most conserved, and the colors in-between designating the corresponding intermediate values of the quantities said.

Six viral capsids are selected as examples from the dataset, with each viral capsid colored by three different rainbow color schemes according to evolutionary or structural features (Fig 1), which are (1) the conservation scores, (2) the centroid-distances (or c-distances) and (3) the WCNs. It is obvious that all three surface representations of each capsid look very similar to each other (a particular example will be discussed in more detail later). It is obvious that conservation scores, WCNs and c-distances appear to have a close link between them. Since conservation scores are derived from aligning homologous sequences, while c-distances and WCNs are derived from a Cα backbone, this indicates that there seems to have a close link between conservation level and structural features. The complete graphics representations of 51 capsids are shown in S1 Fig.

**Fig 2:**
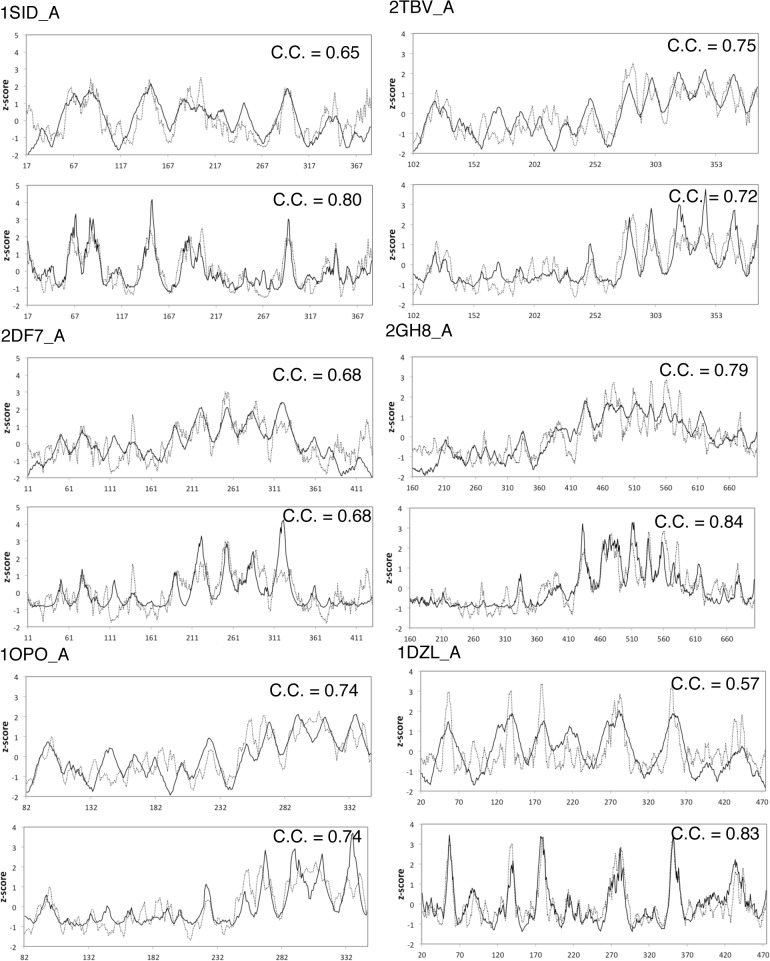
Comparison of profiles. Each plot shows the radius distribution profile (solid line) and conservation profile (dotted line) (the top plot under the PDB ID) and the WCN profile (solid line) and the conservation profile (dotted line) (the bottom plot under the PDB ID). The Pearson’s correlation coefficient is shown on each plot.

To have a quantitative comparison between models, we compute the three types of profiles of the viral proteins of the capsids, shown in Fig 2. The similarities between the sequence conservation profiles and the c-distance profiles or the packing density profiles are impressive, with the Pearson's correlation coefficient between them ranging from 0.65 to 0.84. So now, we have three types of profiles at hand, one from sequence and the other two derived from structure, signifying different biological meanings, and yet we can put them side-by-side for direct comparison. This leads to an interesting conjecture that *the positions of Cα atoms* (hence, no explicit sequence information) contain conservation information comparable with that contained in homologous sequences. For the 51 capsids of the dataset, the average correlation coefficients of the centroid model and the packing density model with the sequence conservation profile for 51 capsids are 0.51 and 0.56, respectively. The complete profiles of 51 capsids are shown in S2 Fig.

**Fig 3:**
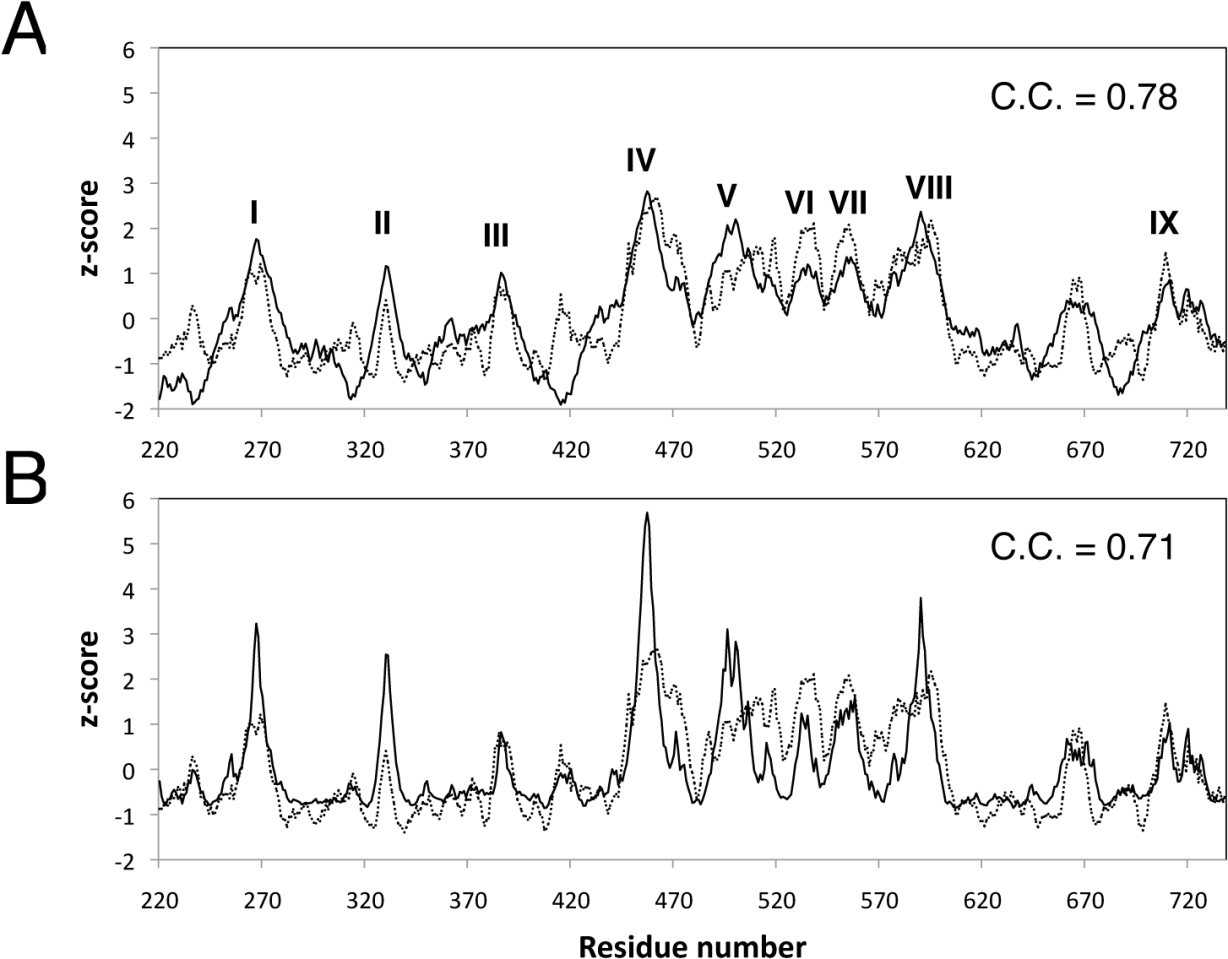
Comparison of the profiles of the viral protein of AAV8 capsid. (A) The c-distance profile (solid line) compared with the sequence conservation profile (dotted line); The variable loop regions I-IX are also labeled. (B) The WCN profile (solid line) compared with the sequence conservation profile. The correlation coefficients are shown.

**Fig 4:**
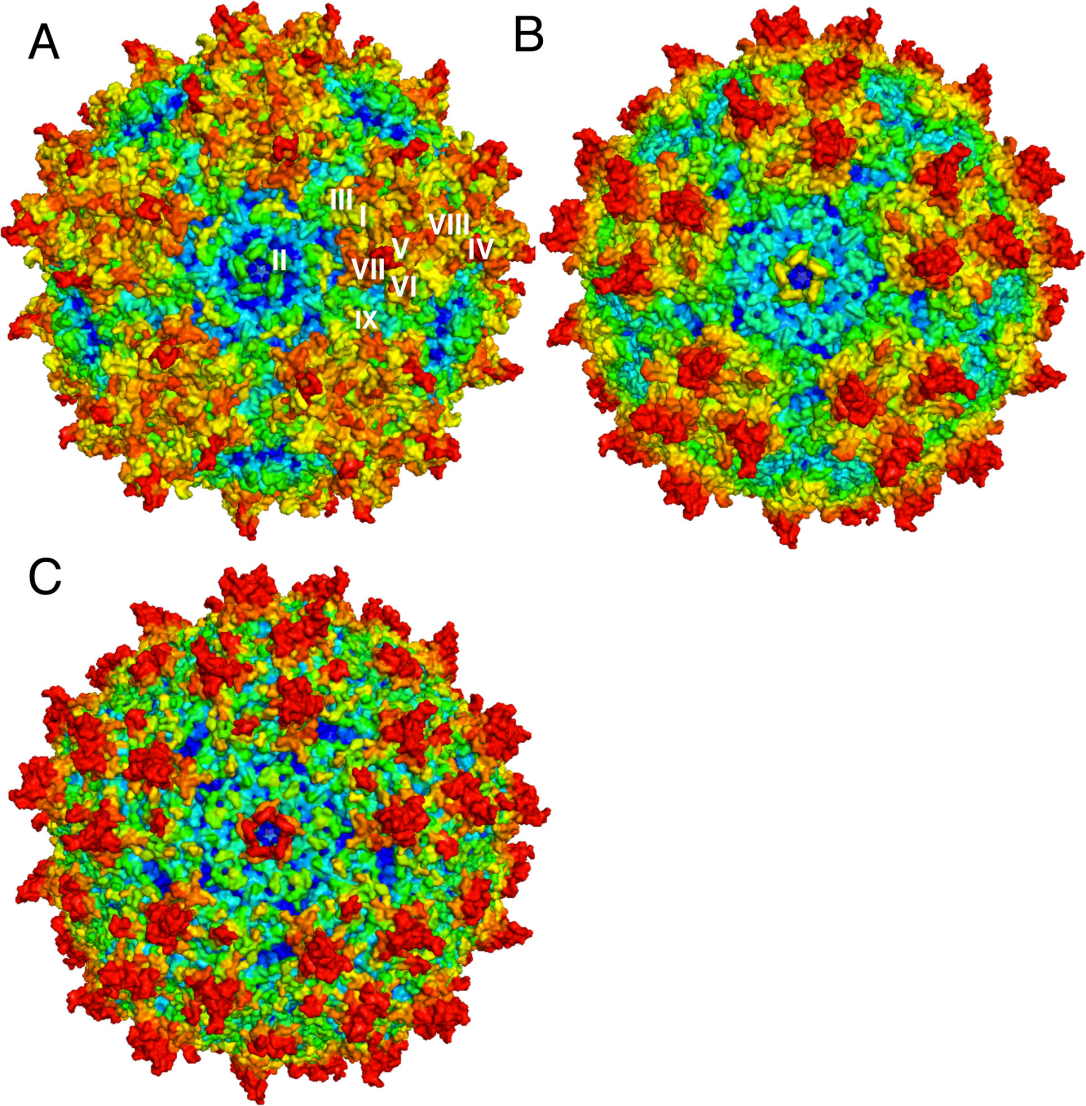
The capsid crystal structure of the AAV serotype 8 (AAV8) in three surface representations. (**A**) the conservation-colored structure, on which the variable loop regions I-IX are labeled, (**B**) the c-distance-colored structure and (**C**) the WCN-colored structure.

It will be instructive to look at a specific example in more details. Adeno-associated viruses (AAVs) have been studied as possible gene therapy vectors to deliver corrective genes into target cells[29–32]. The capsid crystal structure of the AAV serotype 8 (or AAV8) has been determined to 2.6 Å (PDB ID: 2QA0)[30]. Its capsid shell contains 60 structural subunits, each comprising three viral proteins, VP1, VP2 and VP3, all of which are translated from the same mRNA[30]. It is noted that the AAV8 capsid contains 9 variable loop regions (I-IX)[29], which are not only responsible for causing local topological difference from other AAVs, but also are associated with affecting receptor recognition, transduction, and capsid antibody reactivity. The c-distance profile and the WCN profile correctly reproduce 9 variable regions (Fig 3). Both profiles correlate with the sequence conservation profile with excellent Pearson's correlation coefficients of 0.78 and 0.71, respectively. In Fig 4, we show the surface representations of the AAV8 capsid colored according to conservation and structural features, respectively. We label on the conservation-colored surface representation of the variable loop regions, which are easily recognizable as patches or patterns of distinct colors. For example, the variable region II appears as a pentameric form with fivefold symmetry surrounding the channel[31–33].

### On the relation between the centroid model and the packing density model

**Fig 5:**
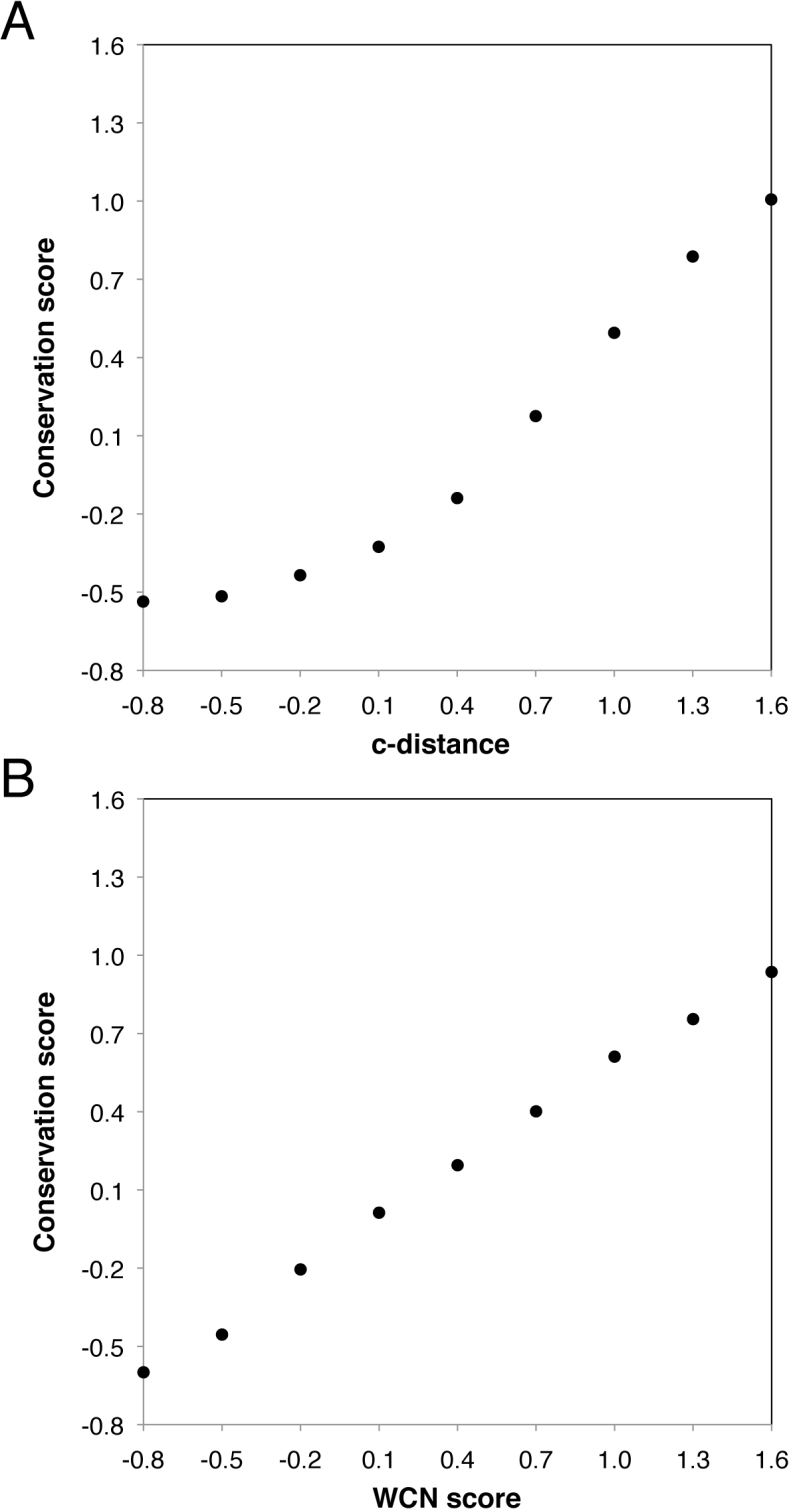
Plots showing different structural features against conservation score. **(A)** the scatter plot of conservation score vs. c-distance; (B) the scatter plot of conservation score vs. WCN score. Note that these quantities are all normalized (see Methods).

In Fig 5, we examine the average trends of c-distances and WCNs along the conservation score for 51 capsids. This is done by collecting the said values (c-distances or WCNs) falling into a specific conservation-bin and averaging them over the residues in that bin. The bin-size Δ*z* is 0.3. The conservation score exhibits a linear behavior with the c-distance for *z*_*r*_ > 0.4, but approaches a plateau-value when the c-distance gets smaller. Therefore, the centroid model is expected to work generally well for residues lying closer to the outer surface of the capsid shell, but, as residues getting closer to the inside surface of the capsid, it will tend to overestimate the conservation levels of those residues. On the other hand, the conservation score shows an excellent linear relationship with the WCN with a correlation *r* ^2^ = 0.997.

**Fig 6:**
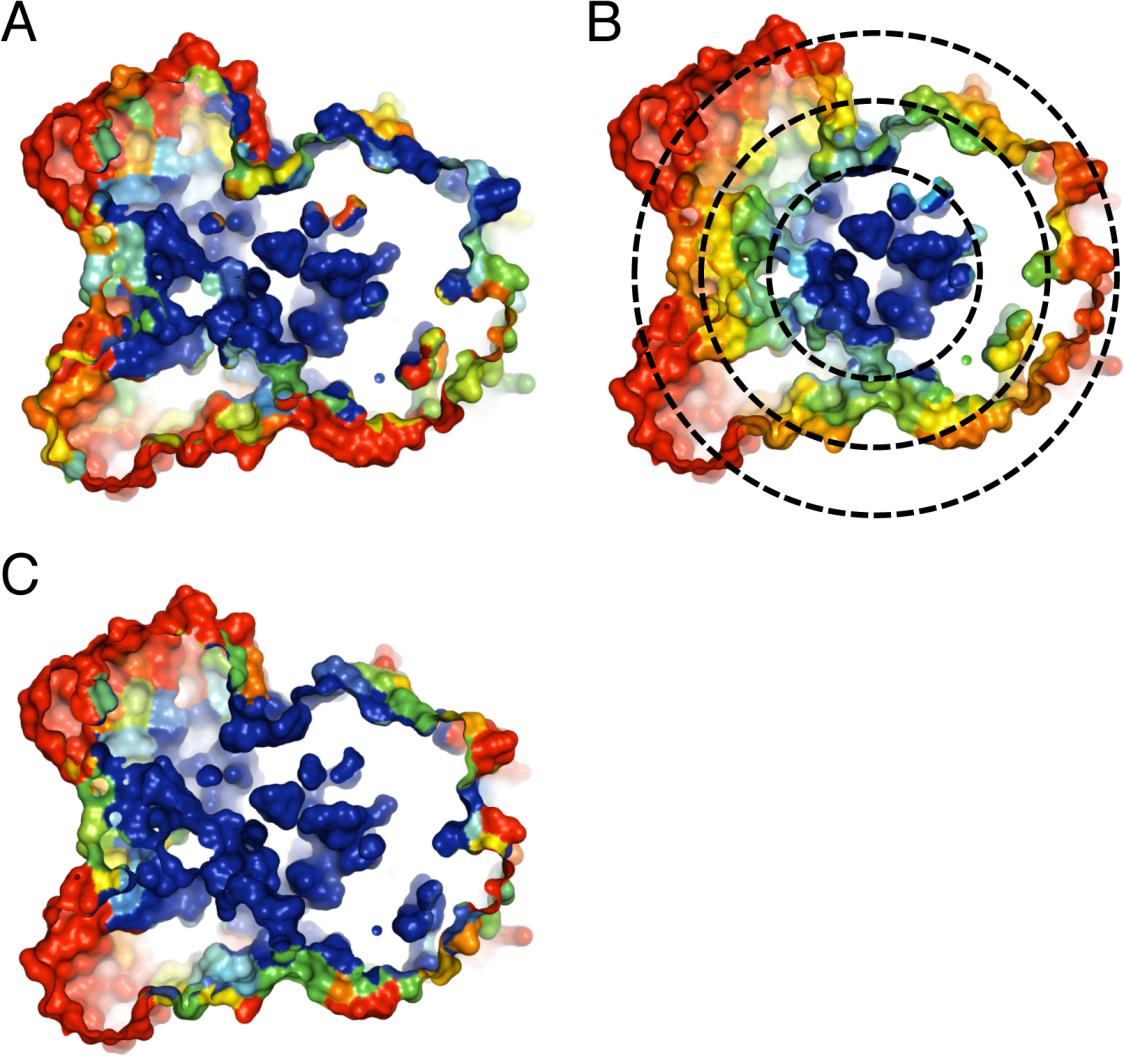
Three surface representations of the structure of 2,4-dienoyl-CoA reductase (PDB ID: 1PS9). Each structure is cut with a clip plane cut through the middle of the structure, showing the core of the protein: (A) the conservation-colored structure; (B) the c-distance-colored structure, together with concentric circles in dotted line with varying radii centered at the centroid; (C) the WCN-colored structure.

The centroid model is an isotropic model (i.e., it is independent of orientations); hence, the centroid model is expected to work well for spherical (or nearly) structures such as icosahedral capsids, as indeed is what we found. But it may not work as well for non-spherical structures. Fig 6 shows such an example. A 2,4-dienoyl-CoA reductase from *E*. *coli* (a non-viral protein)[34] is shown in three models, with a clip plane cut through the middle of the structure, exposing the residues in the core of the protein. The distribution of conservation levels (Fig 6A) is clearly not isotropic as predicted by the centroid model (Fig 6B). But it agrees excellently with the packing density model (Fig 6C).

## Discussion

Considering their simplicity, the structure-based models (i.e., the centroid model and the packing density model), are surprisingly successful in deriving evolutionary information from capsid structures comparable to that from sequence alignment. Both models are based on the idea that the sequence conservation profile is governed by the variations in either c-distance or packing density. The site-specific conservation is usually thought of from an evolutionary viewpoint. It is typically inferred from a multiple sequence alignment of homologous sequences. Numerous methods[35] for detecting residue conservation have been proposed, based on various considerations such as symbol frequency, stereochemical property, mutation data or phylogenetic relationships, though none has currently emerged as a generally accepted standard [36]. By relating residue conservation directly to structural features, the structure-based models allow us to examine residue conservation from a structural viewpoint as well as from an evolutionary viewpoint.

As demonstrated here, variations in residue conservation in capsids relate well to structural features such as the distribution of residues in terms of their separations away from the capsid centroid or spatial inhomogeneities in packing density. By incorporating these features, our models help shed light on the structural basis of residue conservation. On the other hand, the success of these models implies that protein structure can provide information about the level of residue conservation independent of that derived from sequence alignment. Further insight into residue conservation may come from cases where predictions are least accurate; for example, some discrepancies in the packing density model have been attributed to missing binding partners[13,14]. The accuracy of our models might be improved by resorting to an all-atom model, by using weights for different atom types, or by considering binding molecules, though truly quantitative accuracy probably cannot be anticipated from such simple models.

Though conserved residues are usually found close to the protein centroid, things become complicated for non-globular structures like multi-domain proteins or protein complexes[11,12,37,38]. For example, for a structure of several domains, it happens that for some proteins one should compute one centroid for each domain, while for others one need to compute only one centroid for the whole structure[39]. The same dilemma also occurs for protein complexes–for some complexes, one should compute one centroid for each subunit of the complex, while for others one need to compute only one centroid for the complex as a whole[39]. On the other hand, the packing density (or the WCN) can be applied to relating residue conservation in a much more straightforward way than the c-distance. Since protein centroids are usually close to the regions of high packing density[39,40], the centroid model could be seen as an approximation to the WCN model for more symmetrical structures. Indeed, The WCN has been shown to be a much better structure feature to predict catalytic residues than the centroid model [21,24].

Echave et al. [41] noticed that the flexibility of protein backbone, described in terms of atomic mean squared displacements or B-factors, is conserved at family and superfamily levels. Though B-factors are usually interpreted in terms of dynamics, they are in fact static equilibrium properties[42], and thus can be determined from structures without invoking motion. Indeed, studies[22,37,38,43] showed that the B-factors profiles are quantitatively linked to c-distances [37,38] and packing density[22,43]. Based on these studies together with ours, residue rigidity and residue conservation are expected to closely relate to each other.

Therefore, there is a definite relation between the conservation level of residues and their motional thermal fluctuations. Interestingly, this relation can be best explained in terms of Warshel's pre-organization theory[44–46], a theory explaining how enzymes work. Its main idea is that the catalytic residues, being optimized through evolution, are pre-organized in such way that they will remain in similar conformations in both reactant and transition states, thus reducing reorganization energy, to accelerate chemical reactions. There are also reports that residues involved in ligand binding usually have smaller B-factors (i.e., more rigid)[23]. The relationship between packing density and residue conservation may relate to the motional thermal fluctuations or the rigidity of the residues that may be involved in the molecular interactions such as enzymatic reactions, ligand binding or structural packing.

In summary, we show that different profiles, derived from different sources, thus subjecting to different interpretations, can be very closely associated with each other. This means that, if looked upon from a reverse viewpoint, one single profile may be interpreted in different ways. For example, given a conservation profile of a protein sequence, biologists can interpret it in terms of packing density, c-distances or protein flexibility besides conservation, thus gaining a deeper understanding of that protein.

## Supporting Information

S1 Fig The capsid crystal structures of viruses in three surface representations. The conservation surface representation is shown on the left, the centroid surface representation in the middle, and the WCN surface representation on the right.
(PDF)

S2 Fig Comparison of different profiles. Comparison of the radius distribution profile (solid line) and conservation profile (dotted line) (the top plot under the PDB ID) and the WCN profile (solid line) and the conservation profile (dotted line) (the bottom plot under the PDB ID).
(PDF)

S1 Table 51 capsid viruses. (PDF)
