# Supplementary material for: Sequence Conservation, Radial Distance and Packing Density in Spherical Viral Capsids
Source: PLoS One. 2015 Jul 1;10(7):e0132234. doi: 10.1371/journal.pone.0132234 (PMC4488880; doi:10.1371/journal.pone.0132234)
Supplement: S1 Fig — The conservation surface representation is shown on the left, the centroid surface representation in the middle, and the WCN surface representation on the right. (PDF) [file pone.0132234.s001.pdf]

Figure S1. The capsid crystal structures of viruses in three surface representations. The conservation surface representation is shown on the left, the centroid surface representation in the middle, and the WCN surface representation on the right.

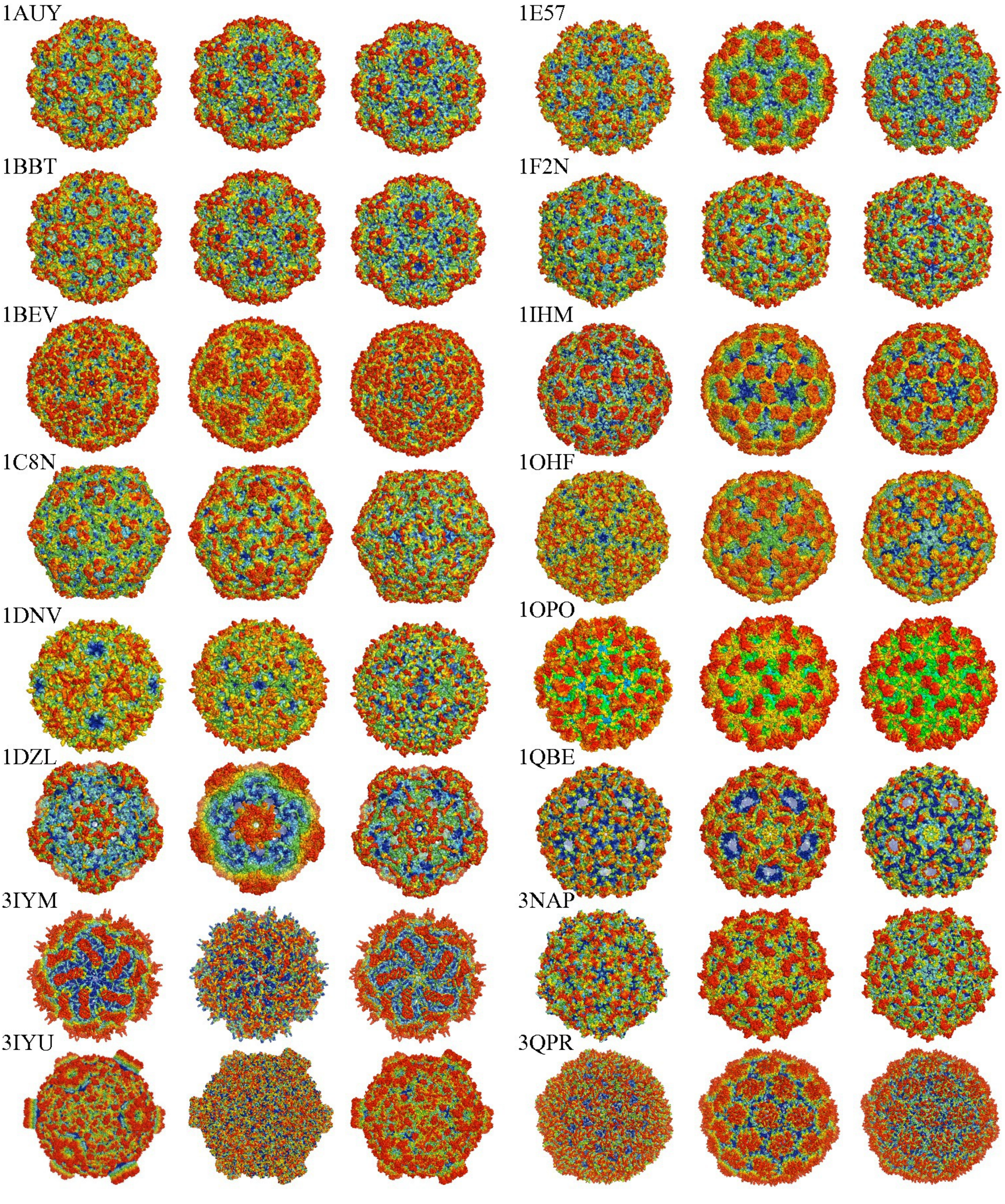

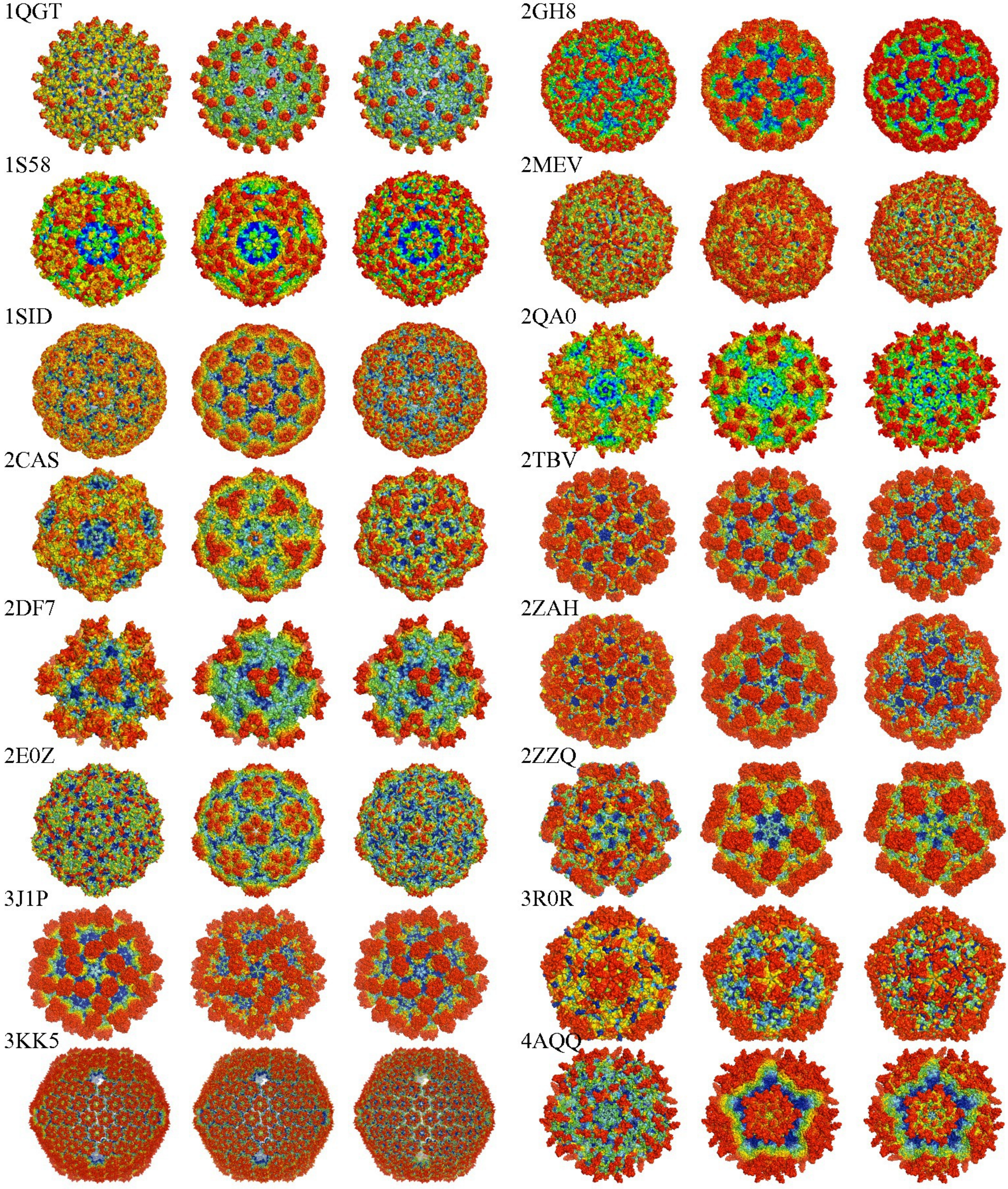
1QGT
1S58
1SID
2CAS
2DF7
2E0Z
3J1P
3KK5
2GH8
2MEV
2QA0
2TBV
2ZAH
2ZZQ
3R0R
4AQQ
