# Supplementary material for: Sequence Conservation, Radial Distance and Packing Density in Spherical Viral Capsids
Source: PLoS One. 2015 Jul 1;10(7):e0132234. doi: 10.1371/journal.pone.0132234 (PMC4488880; doi:10.1371/journal.pone.0132234)

Figures S2. Comparison of different profiles. Comparison of the radius distribution profile (solid line) and conservation profile (dotted line) (the top plot under the PDB ID) and the WCN profile (solid line) and the conservation profile (dotted line) (the bottom plot under the PDB ID).

1AL0_1

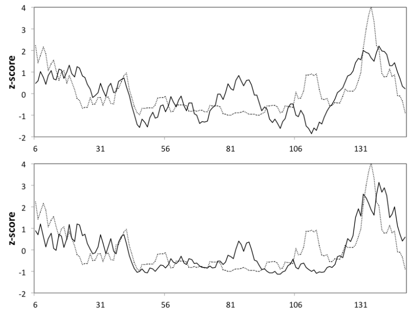

1BEV_3

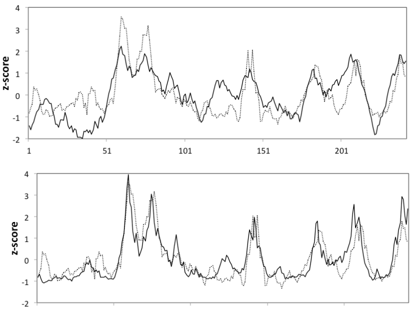

1DZL_A

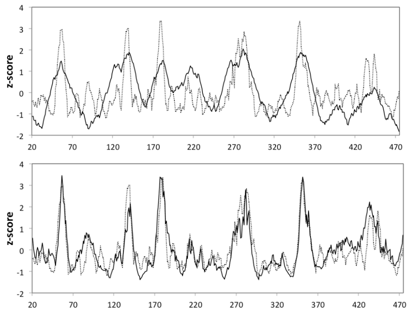

1AUY_A

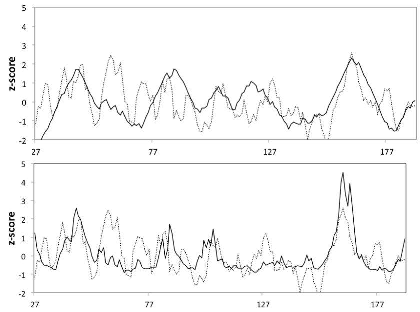

1BMV_1

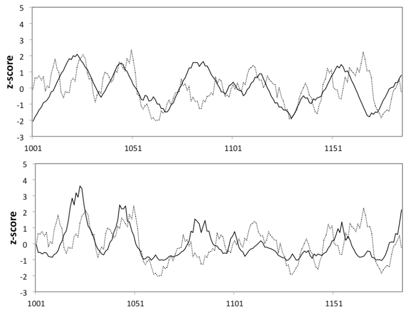

1E57_A

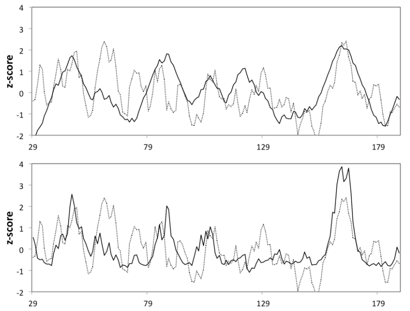

1B35_B

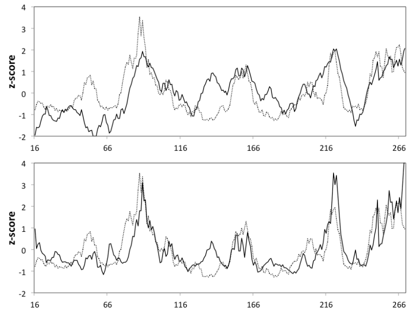

1C8N_A

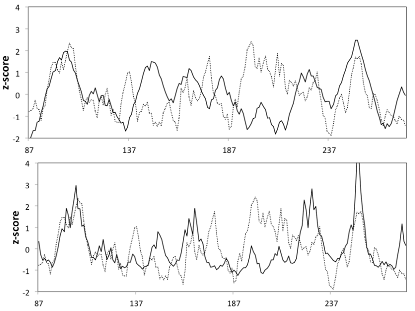

1F2N_A

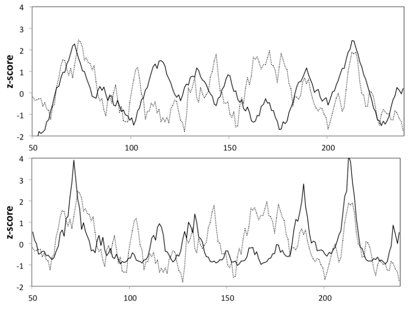

1BBT_1

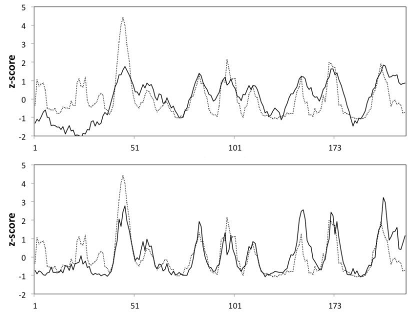

1DNV_A

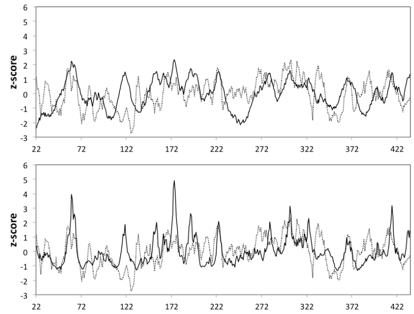

1IHM_A

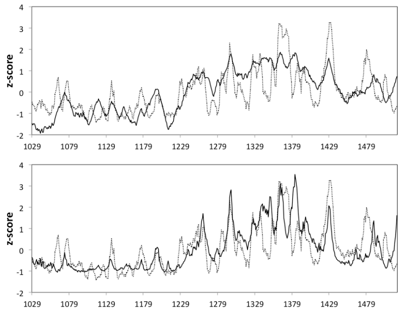

1M06_G

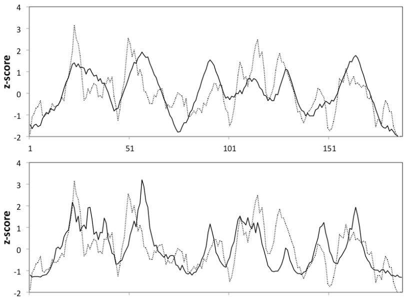

1QGT_C

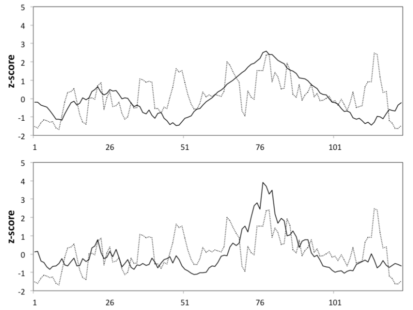

2BTV_C

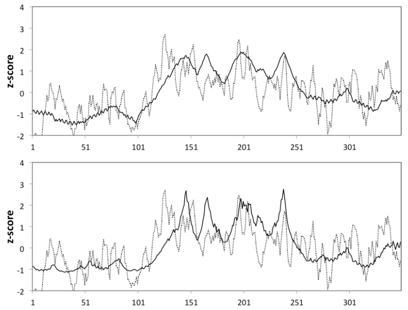

1OHF_A

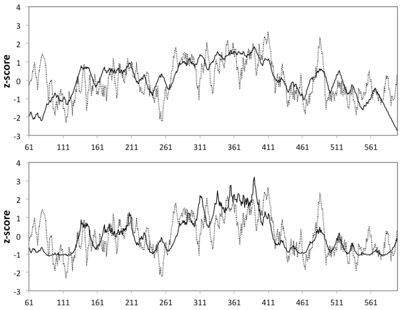

1S58_A

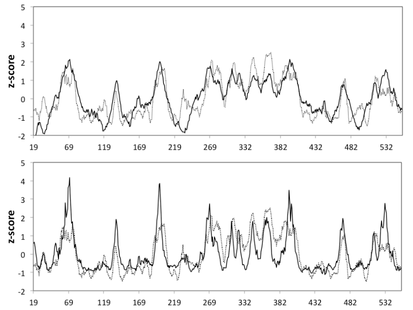

2CAS_A

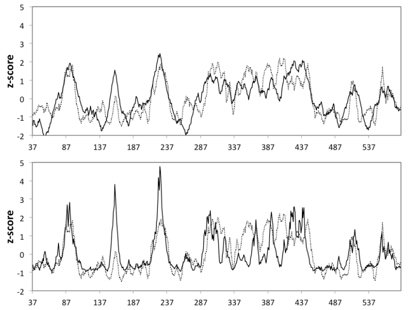

1OPO_A

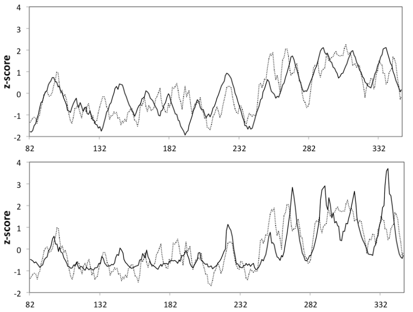

1SID_A

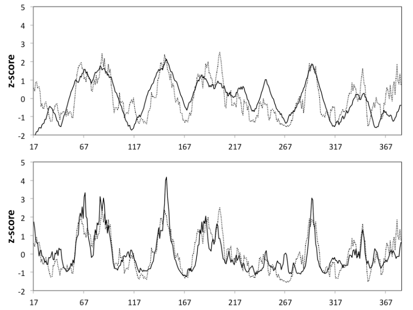

2DF7_A

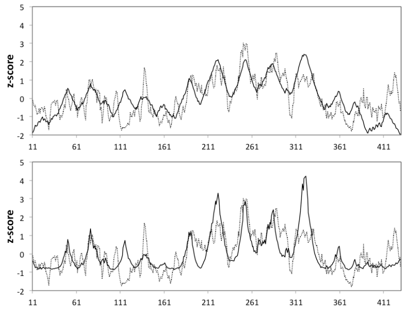

1QBE_A

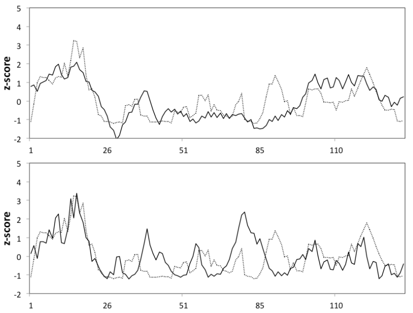

1VSZ_A

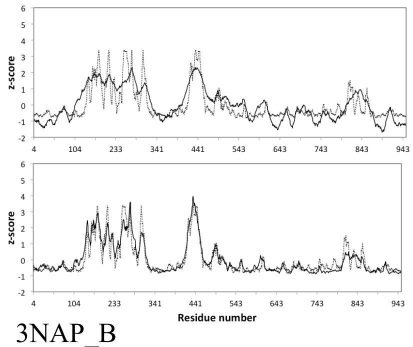

2E0Z_A

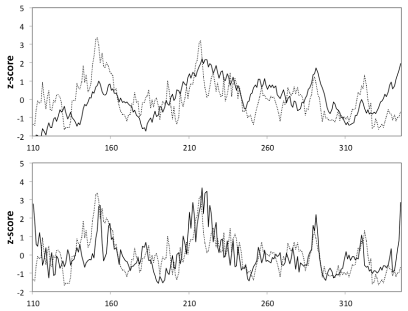

3J1Q_A

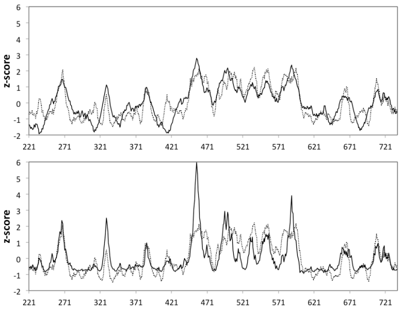

3NAP_B

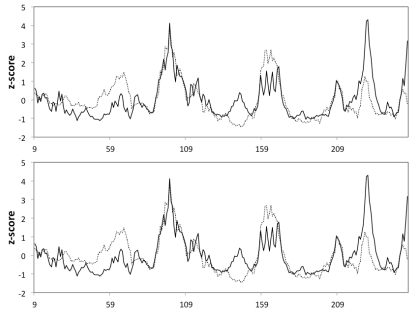

4AN5_A

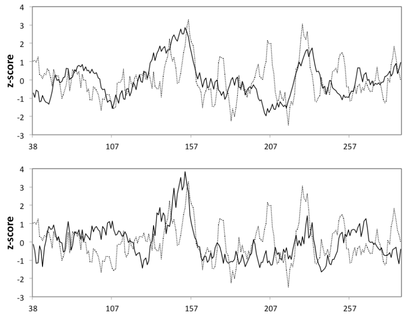

2GH8_A

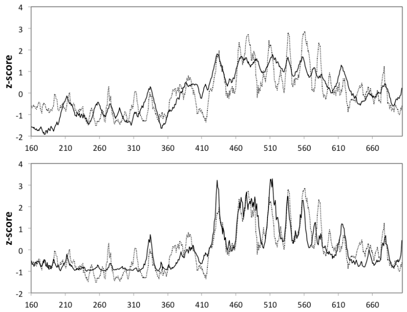

2VF9_A

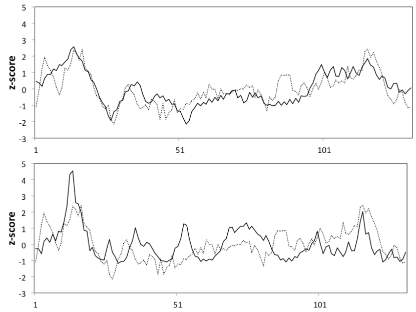

2ZZQ_A

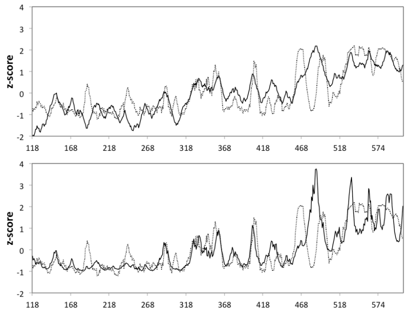

2MEV_1

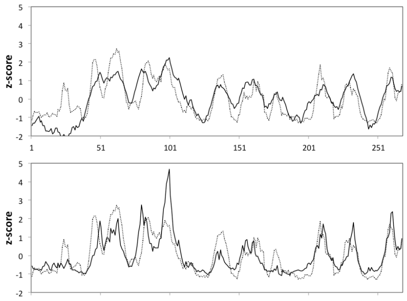

2WFF_1

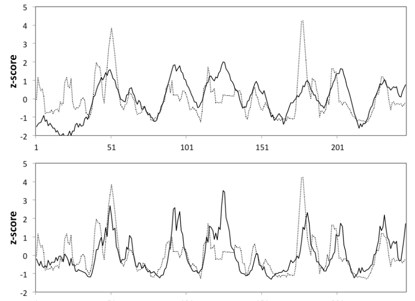

3CJI_C

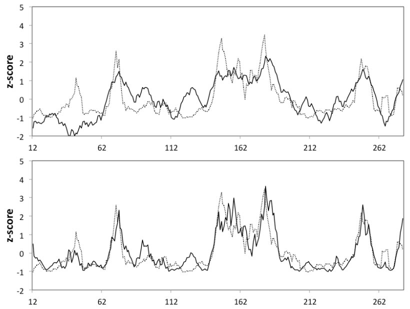

2QA0_A

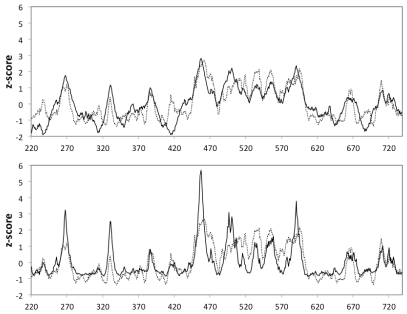

2X8Q_A

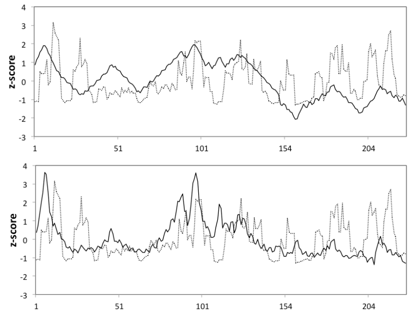

3DPR_E

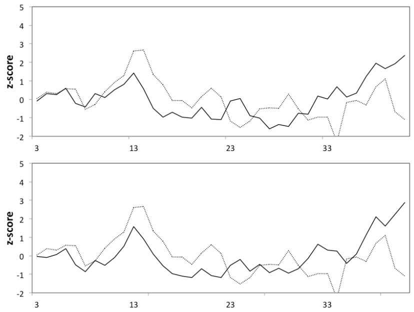

2TBV_A

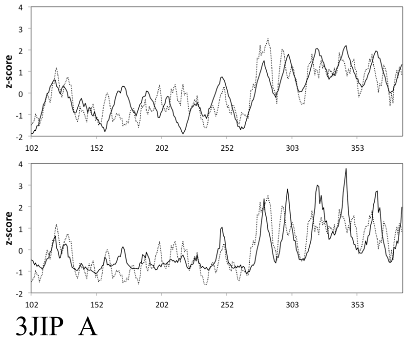

2ZAH_A

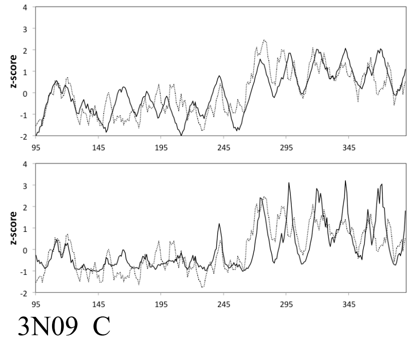

3IYM_
A

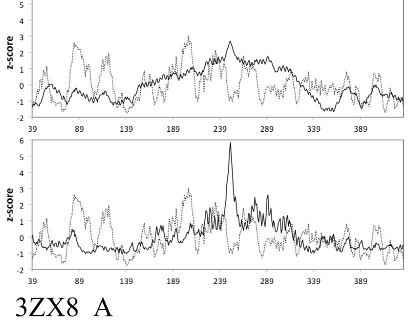

3JIP_A

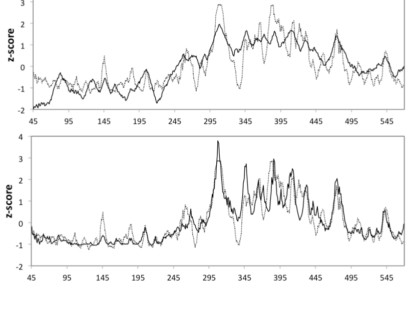

3N09_C

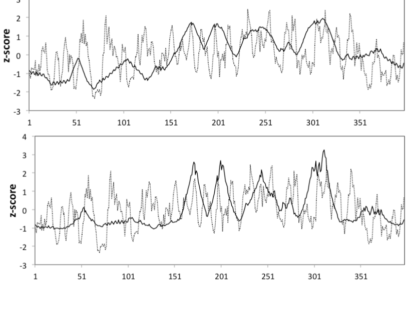

3ZX8_A

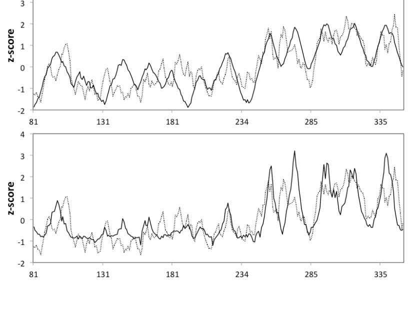

3IYO_A

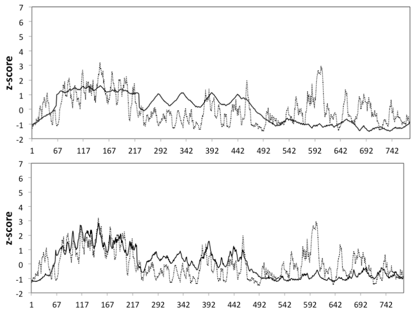

3J40_H

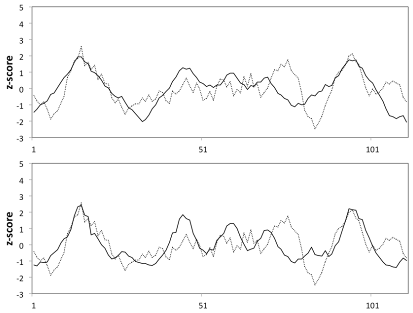

3QPR_A

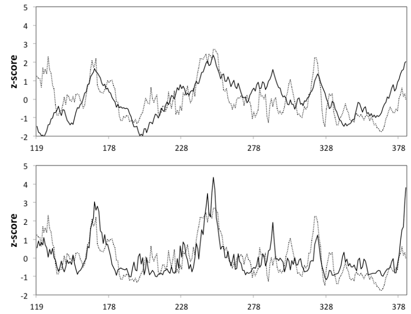

3IZX_D

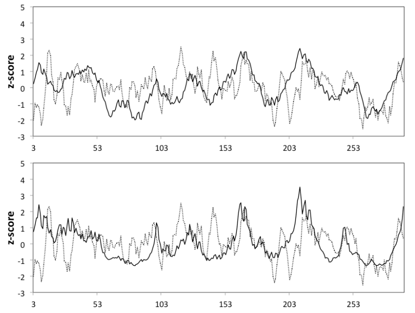

3KK5_A

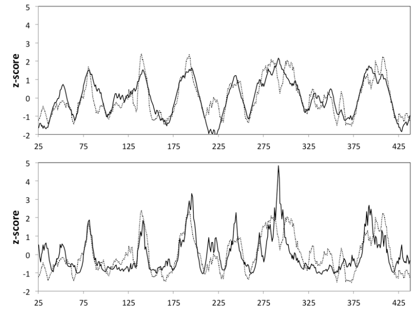

3R0R_A

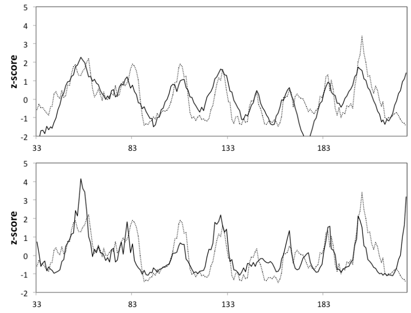

4AQQ_A

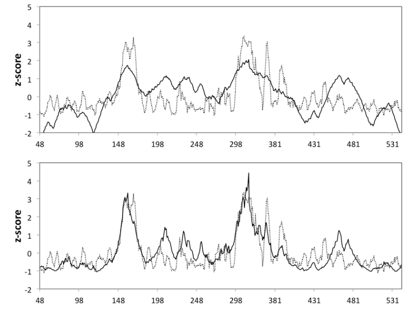

4FTS_A

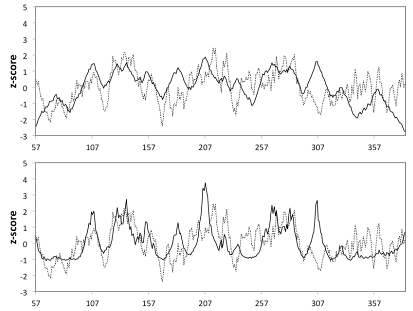

4G0R_A

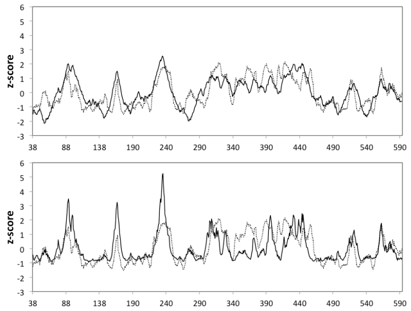

Supplement: S2 Fig — Comparison of the radius distribution profile (solid line) and conservation profile (dotted line) (the top plot under the PDB ID) and the WCN profile (solid line) and the conservation profile (dotted line) (the bottom plot under the PDB ID). (PDF) [file pone.0132234.s002.pdf]
