# Supplementary material for: Sequence Conservation, Radial Distance and Packing Density in Spherical Viral Capsids
Source: PLoS One. 2015 Jul 1;10(7):e0132234. doi: 10.1371/journal.pone.0132234 (PMC4488880; doi:10.1371/journal.pone.0132234)
Supplement: S1 Table — (PDF) [file pone.0132234.s003.pdf]

Table S1. 51 capsid viruses[1]

PDB ID	T number	Number of subunits	Average radius (Å)	Host	Family	Genus
1AL0	1	420	173	Bacteria	Microviridae	Microvirus
1AUY	3	180	152	Plant	Tymoviridae	Tymovirus
1B35	pT3	240	160	insect	Dicistroviridae	Cripavirus
1BBT	pT3	240	149	Human	Picornaviridae	Aphthovirus:
1BEV	pT3	240	154	Animal	Picornaviridae	Enterovirus
1BMV	pT3	120	150	Plant	Comoviridae	Comovirus
1C8N	3	180	155	Plant	Tombusviridae	Necrovirus
1DNV	1	60	130	Insect	Parvoviridae	Densovirus
1DZL	1	60	158	Human	Papillomaviridae	Alphapapillomavirus
1E57	3	180	156	Plant	Tymoviridae	Tymovirus
1F2N	3	180	150	Plant	Sobemoviridae	Sobemovirus
1IHM	3	180	199	Animal	Caliciviridae	Norovirus
1M06	1	180	170	Bacteria	Microviridae	Microvirus
1OHF	4	240	211	Animal	Tetraviridae	Omegatetravirus
1OPO	3	180	176	Plant	Tombusviridae	Carmovirus
1QBE	3	180	143	Bacteria	Leviviridae	Allolevivirus
1QGT	4	240	175	Human	Hepadnaviridae	Orthohepadnavirus
1S58	1	60	135	Human	Parvoviridae	Parvovirus
1SID	7d	360	252	Animal	Polyomaviridae	Polyomavirus
1VSZ	pT25	1200	458	Human	Adenoviridae	Adenoviridae
2BTV	13	900	348	Animal	Reoviridae	Orbivirus
2CAS	1	60	138	Animal	Parvoviridae	Parvovirus
2DF7	1	60	135	Animal	Birnaviridae	Avibirnavirus
2E0Z	2	180	185	Bacteria	Thermococcaceae	Pyrococcus
2GH8	3	180	206	Animal	Caliciviridae	Vesivirus
2MEV	pT3	240	154	Human	Picornaviridae	Cardiovirus
2QA0	1	60	137	Human	Parvoviridae	Densovirus
2TBV	3	180	173	Plant	Tombusviridae	Tombusvirus
2VF9	3	180	139	Bacteria	Leviviridae	Leviviridae
2WFF	pT3	240	154	Animal	Picornaviridae	Aphthovirus
2X8Q	1	60	120	Animal	Retroviridae	Alpharetrovirus
2ZAH	3	180	172	Plant	Tombusviridae	Carmovirus
2ZZQ	1	60	130	Animal	Hepeviridae	Hepevirus
3CJI	pT3	240	157	Animal	Picornaviridae	Senecavirus
3DPR	pT3	300	167	Human	Picornaviridae	Enterovirus
3IYM	2	120	176	Fungi	Partitiviridae	Partitivirus
3IYU	13	960	484	Human	Reoviridae	Rotavirus
3IZX	1	300	299	Insect	Reoviridae	Cypovirus
3J1P	3	180	216	Animal	Caliciviridae	Lagovirus
3J1Q	1	60	138	Animal	Parvoviridae	Dependovirus
3J40	7	840	333	Bacteria	Podoviridae	Epsilon15-like
3KK5	27	780	370	Protozoa	Mimiviridae	Satellite
3N09	13	900	352	Animal	Reoviridae	Rotavirus
3NAP	pT3	180	164	Insect	Dicistroviridae	Cripavirus
3QPR	7	420	254	Bacteria	Siphoviridae	Lambda-like viruses
3R0R	1	60	99	Animal	Circoviridae	Circoviridae
3ZX8	3	180	172	Plant	Tombusviridae	Carmovirus
4AN5	7I	420	331	Bacteria	Siphoviridae	Lambda-like viruses
4AQQ	1	60	146	Human	Adenoviridae	Mastadenovirus
4FTS	3	180	168	Insect	Nodaviridae	Alphanodavirus
4G0R	1	120	140	Human	Parvoviridae	Parvovirus

[1] Carrillo-Tripp, M. et al. VIPERdb(2): an enhanced and web API enabled relational database for structural virology. Nucleic Acids Research **37**, D436-D442, doi:Doi 10.1093/Nar/Gkn840 (2009).
